# Pharmacological Mechanisms of Bile Acids Targeting the Farnesoid X Receptor

**DOI:** 10.3390/ijms252413656

**Published:** 2024-12-20

**Authors:** Youchao Qi, Yonggui Ma, Guozhen Duan

**Affiliations:** 1 College of Agriculture and Animal Husbandry, Qinghai University, Xining 810016, China; 2020990013@qhu.edu.cn; 2 Academy of Agriculture and Forestry Sciences, Qinghai University, Xining 810016, China; 3 Qinghai Plateau Key Laboratory of Tree Genetics and Breeding, Xining 810016, China; 4 Key Laboratory of Medicinal Animal and Plant Resources of Qinghai Tibetan Plateau, Qinghai Normal University, Xining 810008, China; 2025041@qhnu.edu.cn; 5 Academy of Plateau Science and Sustainability, Qinghai Normal University, Xining 810008, China

**Keywords:** BAs, FXR, biological functions, pharmacological mechanisms, signaling pathways

## Abstract

Bile acids (BAs), a category of amphiphilic metabolites synthesized by liver cells and released into the intestine via the bile duct, serve a vital role in the emulsification of ingested fats during the digestive process. Beyond their conventional emulsifying function, BAs, with their diverse structures, also act as significant hormones within the body. They are pivotal in facilitating nutrient absorption by interacting with the farnesoid X receptor (FXR), and they serve as key regulators of lipid and glucose metabolism, as well as immune system balance. Consequently, BAs contribute to the metabolism of glucose and lipids, enhance the digestion and absorption of lipids, and maintain the equilibrium of the bile pool. Their actions are instrumental in addressing obesity, managing cholestasis, and treating diabetes, and are involved in the onset and progression of cancer. This paper presents an updated systematic review of the pharmacological mechanisms by which BAs target the FXR, incorporating recent findings and discussing their signaling pathways in the context of novel research, including their distinct roles in various disease states and populations. The aim is to provide a theoretical foundation for the continued research and clinical application of BAs.

## 1. Introduction

Bile acids (BAs) are stored in the gallbladder of animals and are a crucial component of bile, being signaling molecules and inflammatory agents that rapidly activate nuclear receptors and cell signaling pathways that regulate lipid, glucose, and energy metabolism. They are synthesized from cholesterol in the liver with the assistance of the gut microbiota and are primarily found in the liver and intestines [1,2,3]. BA metabolism is a sophisticated and intricate process that extends beyond the liver into the intestines, where a diverse array of secondary and tertiary BA metabolites are produced through enterohepatic circulation. These metabolites, derived from primary BAs synthesized in the liver, play essential roles in digestion, the absorption of lipids, and the regulation of various metabolic pathways [2]. Secondary BAs and tertiary BAs play crucial physiological roles in the body. Firstly, they are essential for lipid digestion and absorption. Acting as natural detergents, they emulsify dietary fats into smaller droplets, increasing the surface area available for pancreatic lipases to break down fats into fatty acids and glycerol. This process is fundamental for the efficient absorption of lipids and fat-soluble vitamins in the small intestine, which is vital for maintaining normal physiological functions and energy metabolism [4,5]. Secondly, these BAs are involved in cholesterol metabolism regulation. They provide a major excretory route for cholesterol, as cholesterol is converted into BAs in the liver and then excreted into the intestine. By doing so, they help maintain cholesterol homeostasis in the body, preventing the accumulation of excessive cholesterol which could lead to atherosclerosis and other cardiovascular diseases [6]. In addition, secondary and tertiary BAs also contribute to the regulation of energy metabolism. They can activate receptors, such as the farnesoid X receptor (FXR) and the takeda G protein-coupled receptor 5 (TGR5), which in turn regulate the expression of genes involved in glucose and lipid metabolism. For example, activation of the FXR can influence the synthesis and secretion of insulin, while TGR5 activation can stimulate the release of glucagon-like peptide-1 (GLP-1), thereby affecting blood glucose levels and energy expenditure [7,8]. Moreover, they play a role in maintaining intestinal barrier function. They help to strengthen the tight junctions between intestinal epithelial cells, reducing intestinal permeability and preventing the translocation of harmful substances and bacteria from the intestinal lumen into the bloodstream. This is crucial for protecting the body from infections and maintaining gut health [9]. Furthermore, secondary and tertiary BAs have immunomodulatory effects. They can interact with immune cells in the intestine, influencing the balance between pro-inflammatory and anti-inflammatory responses. For instance, some secondary BAs can promote the generation of regulatory T cells, which are important for maintaining immune tolerance and preventing excessive inflammation in the gut [10,11]. Finally, these BAs also have an impact on gut microbiota composition. They can selectively promote the growth of certain beneficial bacteria while inhibiting the growth of pathogenic bacteria, thereby maintaining a healthy gut microenvironment. The gut microbiota, in turn, can further metabolize BAs, forming a complex bidirectional interaction that is essential for overall health [12].

**Figure 1:**
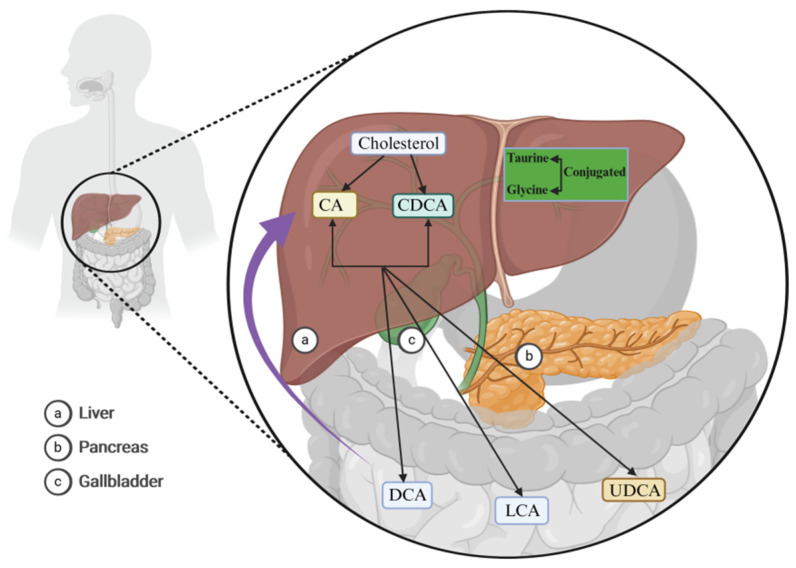
Bile acid (BA) synthesis. In the human liver, BAs are synthesized via the enzymatic catalysis of three cholesterol hydroxylases: cholesterol 7α-hydroxylase (CYP7A1), human sterol 12a-hydroxylase (CYP8B1), and the human sterol 27-hydroxylase gene (CYP27A1), giving rise to cholic acid (CA) and chenodeoxycholic acid (CDCA) (black arrows). They subsequently undergo dehydroxylation at the C7 position of CA and CDCA, resulting in the formation of secondary BAs, such as deoxycholic acid (DCA), lithocholic acid (LCA), and ursodeoxycholic acid (UDCA) (black arrows). These BAs predominantly modulate physiological and pathological processes within the body through hepatointestinal circulation (purple arrow). Abbreviations: BAs, bile acids; CYP7A1, cholesterol 7α-hydroxylase; CYP8B1, human sterol 12a-hydroxylase; CYP27A1, human sterol 27-hydroxylase gene; CA, cholic acid; CDCA, chenodeoxycholic acid; DCA, deoxycholic acid; LCA, lithocholic acid; UDCA, ursodeoxycholic acid.

**Figure 2:**
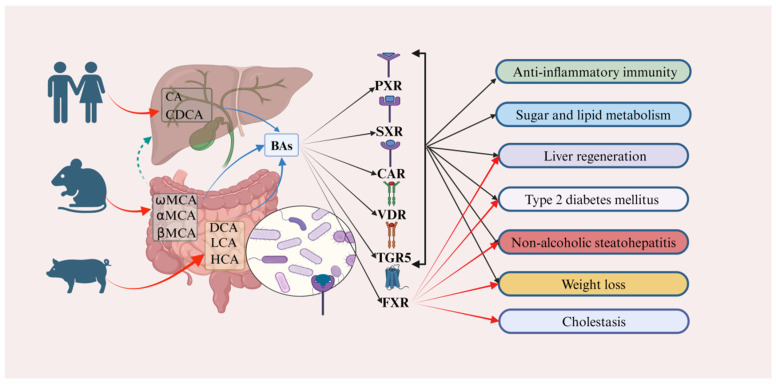
Bile acids (BAs) play a crucial role in various physiological and pathological processes by targeting and mediating diverse receptors. In humans, cholic acid (CA) and chenodeoxycholic acid (CDCA) are predominantly expressed. In contrast, α-muricholic acid (αMCA), β-muricholic acid (βMCA), and ω-muricholic acid (ωMCA) are primarily expressed in mice and rats. Additionally, deoxycholic acid (DCA), lithocholic acid (LCA), and hyocholic acid (HCA) are predominantly expressed in pigs (red curved arrows). These BAs are involved in regulating anti-inflammatory immunity, sugar and lipid metabolism, liver regeneration, type 2 diabetes mellitus (T2DM), non-alcoholic steatohepatitis (NASH), weight loss, and cholestasis, primarily through targeted binding to receptors such as the pregnane X receptor (PXR), the steroid and X-enobiotic receptor (SXR), the constitutive androstane receptor (CAR), the vitamin D receptor (VDR), and the takeda G protein-coupled receptor 5 (TGR5) (black rays arrows and solid black arrows). Moreover, when these BAs target the farnesoid X receptor (FXR), they predominantly contribute to the regulation of liver regeneration, type 2 diabetes mellitus (T2DM), non-alcoholic steatohepatitis (NASH), weight loss, and cholestasis (red rays arrows). The green arrows represents the hepatoenteric circulation. Abbreviations: CA, cholic acid; CDCA, chenodeoxycholic acid; CAR, constitutive androstane receptor; DCA, deoxycholic acid; farnesoid X receptor (FXR); HCA, hyocholic acid; LCA, lithocholic acid; NASH, non-alcoholic steatohepatitis; PXR, pregnane X receptor; SXR, steroid and X-enobiotic receptor; T2DM, type 2 diabetes mellitus; TGR5, takeda G protein-coupled receptor 5; VDR, vitamin D receptor; αMCA, α-muricholic acid; βMCA, β-muricholic acid; ωMCA, ω-muricholic acid.

Studies have shown that, in higher animals, BAs are typically derived from 24-carbon cholanic acids, while in fish, amphibians, and reptiles, they are derived from 27- or 28-carbon phytosterols, indicating that the composition of BAs varies across different animal species [13,14,15]. In humans, the main BAs are cholic acid (CA), chenodeoxycholic acid (CDCA), deoxycholic acid (DCA), and lithocholic acid (LCA) (Figure 1) [16]. In rats and mice, they include CA, alpha-muricholic acid (α-MCA), beta-muricholic acid (β-MCA), and omega-muricholic acid (ω-MCA), while in pigs, they include hyocholic acid (HCA), which is about 25 times more prevalent than in humans. BAs serve as an important internal signaling molecule, interacting with the FXR, pregnane X receptor (PXR), steroid and xenobiotic receptor (SXR), constitutive androstane receptor (CAR), vitamin D receptor (VDR), and the TGR5 [17]. They participate in various physiological and pathological processes, such as anti-inflammatory immunity, sugar and lipid metabolism, liver regeneration, type 2 diabetes mellitus (T2DM), non-alcoholic steatohepatitis (NASH), and weight loss [18,19]. Recent research has highlighted the particularly strong relationship between the FXR receptor and cholestasis, liver regeneration, T2DM, NASH, and weight loss (Figure 2) [11,12,13,14,15,16,17,18,19,20,21,22,23,24].

The FXR receptor, a distinguished member of the nuclear receptor superfamily, is encoded by two genes, FXRα and FXRβ, in humans, under the governance of the nuclear receptor gene NR1H4 [25]. A multitude of ligands have the capacity to activate the FXR receptor, including GSK2324, GW4064, GSK8062, FXR-450, obeticholic acid (6-ethylchenodeoxycholic acid, OCA), taurochenodeoxycholate acid (TCDCA), ursodeoxycholic acid (UDCA), and taurolithocholic acid (TLCA) [25,26]. Notably, BAs serve as the natural activators of the FXR receptor, endowing it with the additional moniker of the BAs receptor. Predominantly expressed in the liver, small intestine, and kidneys, the FXR receptor fulfills a pivotal biological role in the maintenance of cholesterol and BAs homeostasis, achieved through its regulation of sugar and fatty acid metabolic pathways [27].

## 2. BAs Regulate Cholestasis via the FXR

Cholestasis is a complex clinical pathophysiological process characterized by the impaired secretion of bile into the duodenum, which is essential for metabolism. This condition arises from liver cell damage due to early cholestasis, and can progress to liver fibrosis and, ultimately, liver failure. Cholestasis is commonly categorized into two primary types: primary biliary cirrhosis (PBC) and primary sclerosing cholangitis (PSC) [28]. In the case of PBC, the clinical manifestations typically encompass fatigue, jaundice, and itching. A study conducted by Hou and his co-workers on 45 patients with PSC revealed that all patients exhibited significantly increased serum levels of alkaline phosphatase (ALP) and gamma-glutamyltransferase (GCT) [29]. These elevated enzyme levels have been identified as reliable biomarkers for the clinical diagnosis of PBC. Furthermore, Song et al. conducted a clinical serological analysis on 100 patients with PSC, identifying common symptoms such as fatigue, urinary discoloration, and weight loss [30]. Abnormal elevations in serum levels of ALP, aspartate aminotransferase (AST), alanine aminotransferase (ALT), and total bilirubin (TBil) were observed in these patients, suggesting that these markers can also be utilized as diagnostic indicators for PSC [31].

**Figure 3:**
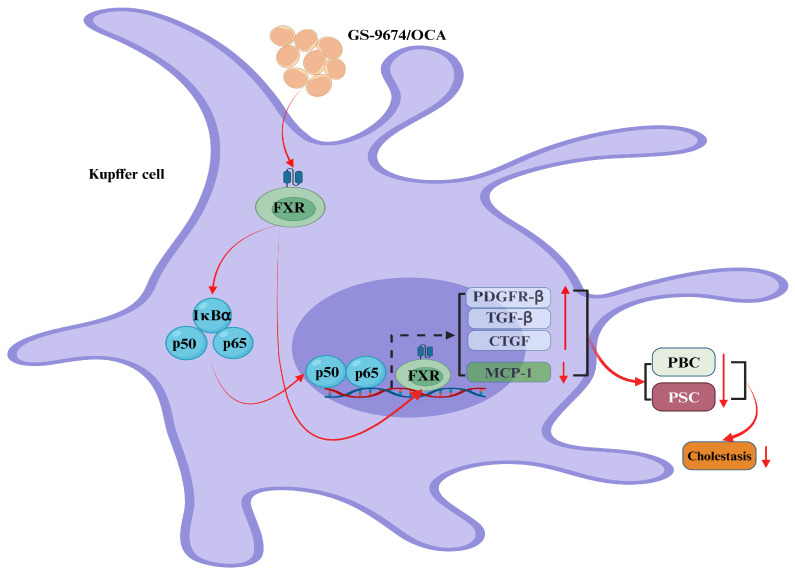
Bile acids (BAs) regulate cholestasis via the farnesoid X receptor (FXR). In Kupffer cells, GS-9674 and obeticholic acid (OCA) exert their effects by activating the cellular FXR receptor, which results in its direct translocation to the nucleus. This process significantly enhances the expression of the platelet-derived growth factor β receptor (PDGFRβ), the transforming growth factor-β (TGFβ), and the connective tissue growth factor (CTGF), while concurrently reducing the levels of the monocyte chem-attractant protein-1 (MCP-1). Consequently, this dual mechanism markedly diminishes the expression of primary biliary cirrhosis (PBC) and primary sclerosing cholangitis (PSC), thereby contributing to the inhibition of biliary tract obstruction. Additionally, GS-9674 and OCA stimulate the cytoplasmic FXR receptor, which in turn triggers the nuclear translocation of the nuclear factor kappa-B (NF-κB) (red rays arrows). Once within the nucleus, NF-κB activates the FXR receptor, further potentiating the expression of the platelet-derived growth factor receptor (PDGFR), the transforming growth factor (TGF), and the CTGF (up-solid red arrows), and concurrently reducing MCP-1 expression (down-solid red arrows). This cascade effectively reduces the expression of PBC and PSC, playing a pivotal role in alleviating biliary tract obstruction (down-solid red arrows). Abbreviations: CTGF, connective tissue growth factor; FXR, farnesoid X receptor; MCP-1, monocyte chem-attractant protein-1; NF-κB, nuclear factor kappa-B; OCA, obeticholic acid; PDGFRβ, platelet-derived growth factor β receptor; PDGFR, platelet-derived growth factor receptor; TGFβ, transforming growth factor-β; TGF, transforming growth factor; PBC, primary biliary cirrhosis; PSC, primary sclerosing cholangitis.

In the year 2018, Gilead Sciences, an American corporation, heralded the discovery that its novel FXR receptor agonist, GS-9674, possesses a remarkable capacity to significantly reduce the levels of ALP, GCT, and TBil. This breakthrough holds promise for the treatment of liver diseases, such as PBC and PSC [32]. Despite their distinct chemical structures, both OCA and GS-9674 function as potent agonists of the FXR receptor, thereby exerting a profound impact on pharmacological outcomes [33]. Within hepatocytes and Kupffer cells (KCs), these agents effectively activate the FXR receptor, leading to a marked inhibition of the nuclear translocation of the nuclear factor kappa-light-chain-enhancer of activated B cells (NF-κB) [34]. Consequently, the expression of the connective tissue growth factor (CTGF), the transforming growth factor-beta (TGF-β), the platelet-derived growth factor beta-receptor (PDGFR-β), and the monocyte chem-attractant protein-1 (MCP-1) is significantly reduced [35,36]. This intricate mechanism of action effectively curtails the production and uptake of BAs within the liver, while concurrently enhancing bile flow. Such actions are instrumental in mitigating the onset and progression of PBC and PSC, offering a beacon of hope for patients afflicted by these challenging conditions (Figure 3).

## 3. BAs Regulate NAFLD via the FXR

BAs play a crucial role in enterohepatic circulation. Disruption of this cycle can lead to the onset and progression of various diseases, such as NASH, cirrhosis, and ulcerative colitis (UC) [37,38,39]. Notably, NASH is involved in the pathogenesis and metabolism of gallstones and hypercholesterolemia. In the context of human-like hydrophobic BA models, the activation of the cytokine-c-Jun N-terminal kinases (JNKs)-mitogen-activated protein kinases (MAPKs) signaling pathway by the FXR receptor emerges as a pivotal mechanism for regulating BA synthesis during acute liver injury, such as that induced by cholesterol [40]. The formation of gallstones is intricately associated with the saturation of cholesterol within the gallbladder and the hydrophobicity of BAs. Notably, obeticholic acid (OA) has been observed to significantly elevate the expression of key enzymes, including the human cytochrome P450 (CYP) 3A4 (CYP3A4), the sulfotransferase family 2A member 1 (SULT2A1), the UDP- glucuronosyltransferase family 2 member B4 (UGT2B4), and the multidrug resistance protein 3 (MDR3), by specifically activating the FXR receptor in human subjects [41,42]. This activation notably reduces the solubility of cholesterol, and concurrently increases the saturation of cholesterol in the gallbladder and the hydrophobicity of BAs. Consequently, OA effectively mitigates the development of gallstones. In obese and diabetic db/db mice, the pharmacological activation of AMPK by BAs, or its overexpression in hepatocytes, inhibited FXR receptor expression and further decreased expression of the hepatocyte nuclear factor 4 (HNF-4), the peroxisome proliferator activated receptor-γ coactivator-1α (PGC-1α) and the forkhead transcription factor Foxo1 (also known as FKHR), significantly inhibiting expression of glucose-6-phosphatase (G6Pase), phosphoenolpyruvate carboxykinase (PEPCK), and fructose 1,6-bis phosphatase (FBP1) to lower blood glucose levels, decreased the free fatty acids (FFA) levels, and increased insulin sensitivity [43,44,45,46].

**Figure 4:**
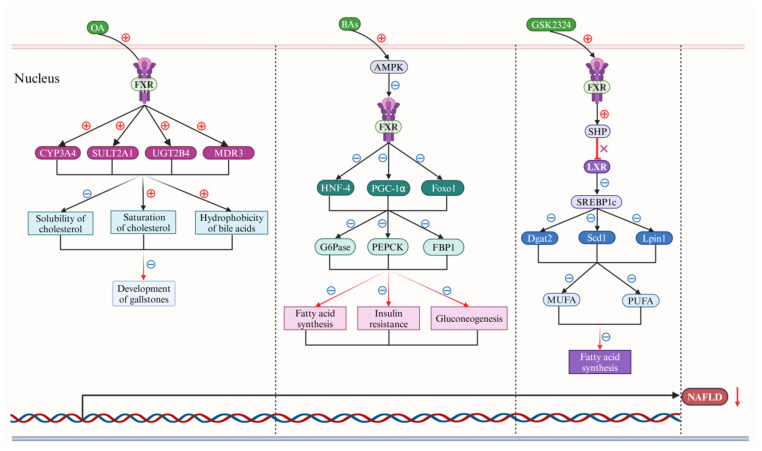
Bile acids (BAs) regulate nonalcoholic fatty liver disease (NAFLD) via the farnesoid X receptor (FXR). In the nucleus of liver cells, obeticholic acid (OA) initially demonstrates a notable reduction in the solubility of cholesterol, a marked enhancement in cholesterol saturation levels, and a significant decrease in the hydrophobicity of BAs (black rays arrows). This is predominantly achieved through the up-regulation of the human cytochrome P450 (CYP) 3A4 (CYP3A4), the sulfotransferase family 2A member 1 (SULT2A1), the UDP glucuronosyl transferase family 2 member B4 (UGT2B4), and multidrug resistance protein 3 (MDR3) expression (solid-black arrows), which is facilitated by the targeted activation of the farnesoid X receptor (FXR), thereby alleviating the formation of gallstones (down-red rays arrows). Subsequently, BAs stimulate mitogen-activated protein kinases (MAPKs) activation significantly, which markedly inhibits expression of the hepatocyte nuclear factor 4 (HNF-4), the peroxisome proliferator-activated receptor-γ coactivator-1α (PGC-1α), and the forkhead transcription factor 1 (Foxo1) by reducing FXR expression (solid-black arrows). This continued down-regulation affects the expression of glucose-6-phosphatase (G6Pase), phosphoenol pyruvate carboxy kinase (PEPCK), and DNA-binding protein phosphatase 1 (DBP1) (solid-black arrows), ultimately leading to a decrease in fatty acid synthesis, insulin resistance, and gluconeogenesis (red rays arrows). Furthermore, GSK2324, a specific agonist for the FXR receptor, enhances the expression of the small heterodimer partner (SHP) by activating the FXR receptor (down-red rays arrows), subsequently blocking liver X receptor (LXR) expression (red terminating line and X). This cascade inhibits the sterol-regulatory element binding protein 1c (SREBP1c) (down-solid black arrows), reducing the expression of diacylglycerol acyltransferase 2 (Dgat2), stearoyl-CoA desaturase 1 (Scd1), and phosphatidic acid phosphohydrolase 1 (Lpin1), and ultimately results in a significant reduction in fatty acid synthesis by down-regulating the expression of monounsaturated fatty acid (MUFA) and polyunsaturated fatty acid (PUFA) (solid black arrows). Collectively, these actions significantly impede the progression of NAFLD (down-solid red arrow). Abbreviations: BAs, bile acids; CYP3A4, human cytochrome P450 (CYP) 3A4; DBP1, DNA-binding protein phosphatase 1; Dgat2, diacylglycerol acyltransferase 2; FXR, farnesoid X receptor; Foxo1, forkhead transcription factor 1; G6Pase, glucose-6-phosphatase; HNF-4, hepatocyte nuclear factor 4; LXR, liver X receptor; lpin1, phosphatidic acid phosphohydrolase 1; MDR3, multidrug resistance protein 3; MAPK, mitogen-activated protein kinase; MUFA, monounsaturated fatty acid; NAFLD, non-alcoholic fatty liver disease; OA, obeticholic acid; PGC-1α, peroxisome proliferator-activated receptor-γ coactivator-1α; PEPCK, phosphoenol pyruvate carboxy kinase; PUFA, polyunsaturated fatty acid; SULT2A1, sulfotransferase family 2A member 1; Scd1, stearoyl-CoA desaturase 1; UGT2B4, UDP glucuronosyl transferase family 2 member B4; SHP, small heterodimer partner. Note: The symbol ⊕ represents upregulation, whereas the symbol ㊀ signifies downregulation.

In the context of lipid disorders, the activation of the FXR receptor through the administration of the FXR agonist GSK2324 effectively reduces the hepatic levels of both mono- and polyunsaturated fatty acids (MUFAs and PUFAs) [47,48]. This reduction is achieved by suppressing the expression of key enzymes involved in fatty acid synthesis, namely stearoyl-CoA desaturase 1 (Scd1), diacylglycerol acyltransferase 2 (Dgat2), and phosphatidic acid phosphohydrolase1 (Lpin1) [47]. Notably, this mechanism operates independently of the regulatory pathways controlled by the small heterodimer partner (SHP) and the sterol regulatory element-binding protein 1c (SREBP1c) by blocking the liver X receptor (LXR), which are pivotal in governing the expression of hepatic lipid genes [48,49]. Furthermore, FXR receptor activation in the intestine contributes to the inhibition of lipid absorption, thereby providing a comprehensive strategy for the management of lipid disorders. Therefore, various BAs exert distinct pharmacological functions by targeting the FXR receptor to regulate non-alcoholic fatty liver disease (NAFLD). Some BAs positively modulate this interaction, while others negatively regulate disease processes. Consequently, the discovery and investigation of numerous BAs that target the FXR receptor could potentially mitigate the onset and progression of diseases, thereby alleviating the suffering of living organisms (Figure 4).

Gender disparities significantly influence the distribution of BAs in NAFLD and alcohol-related liver disease (ALD), as recent studies have accentuated. Fitzinger et al., in their pivotal 2024 investigation, highlighted pronounced sex-specific variations in the BA spectrum within NAFLD, with even more marked differences observed in the BA patterns of ALD patients [50]. These insights suggest that gender may contribute to the progression of liver pathologies by modulating BA synthesis and catabolism. Furthermore, Sheng et al., in their 2017 research, probed the correlation between gender disparities and the incidence of diet-induced fatty liver, as well as the inactivation of the FXR [51]. Their study elucidates the role of gender in the interplay between BAs, gut microbiota, and the onset of fatty liver disease. The impact of sexual dimorphism on BA profiles may be attributed to alterations in sex hormone levels, which are recognized to govern the expression of genes involved in hepatic BA synthesis [52]. For instance, estrogen has been demonstrated to enhance the expression of CYP7A1, a pivotal enzyme in CA biosynthesis, thereby influencing BA production [43]. Moreover, androgens may modulate BA metabolism by acting upon FXR signaling pathways [53]. These hormonal influences likely account for the observed sex-specific BA profiles in NAFLD and ALD. Beyond the realm of sex hormones, gender differences may also correlate with lifestyle choices, dietary behaviors, and genetic predispositions. Certain investigations have revealed distinct BA profiles in female versus male NAFLD patients, which may be associated with a propensity among women to adhere to a lower-fat diet and a healthier lifestyle [54]. Additionally, genetic factors may contribute to the sexual dimorphism, as certain genes involved in BAs metabolism exhibit differential expression between sexes. In the context of FXR signaling, the significance of gender disparities cannot be overlooked. The FXR serves as the principal sensor for BAs, with its activation modulating the synthesis, secretion, and metabolism of these acids. Research indicates that there may be discernible variations in the expression and functionality of the FXR between genders, potentially influencing the signaling and metabolic pathways of BAs [55]. For instance, FXR activation may exert a more potent anti-inflammatory effect in females, whereas in males, it may predominantly regulate energy and lipid metabolism [56]. In conclusion, gender distinctions represent a multifaceted and critical aspect within the realm of BAs and FXR research. Future investigations are imperative to delve deeper into the ways in which gender influences BA metabolism and FXR signaling, as well as the implications of these disparities on the onset and progression of liver diseases. A profound comprehension of these mechanisms will lay the theoretical groundwork for gender-specific interventions in the treatment of NAFLD and ALD, and facilitate the development of more efficacious therapeutic strategies.

## 4. BAs Regulate T2DM via the FXR

Diabetes mellitus (DM), commonly referred to as DM, is a prevalent chronic metabolic disorder that currently affects approximately 540 million individuals globally. Clinically, DM is categorized into two primary types: type 1 diabetes (T1DM) and T2DM, with T2DM comprising over 90% of all diabetic cases. The onset and progression of T2DM are influenced by a multitude of factors, including familial history, age, obesity, and hypertension, typically manifesting in individuals over the age of 35–40 [41,42,43,44,45]. Recent research has illuminated a correlation between T2DM and an augmented risk of various cancers [57,58]. These include a 21% heightened risk for colorectal cancer, a 27% increased risk for lung cancer, a 107% escalated risk for pancreatic cancer, an 85% augmented risk for esophageal cancer, an 18% heightened risk for kidney cancer, a 239% increased risk for liver cancer, a 49% heightened risk for thyroid cancer, a 33% increased risk for gallbladder cancer, a 26% heightened risk for breast cancer, and a 26% increased risk for endometrial cancer [59,60,61]. At present, the most effective treatment for T2DM encompasses a combination of a healthy lifestyle and pharmacological intervention. In an era characterized by rapid technological advancements and an accelerated pace of life, it has become increasingly challenging for individuals to adopt and sustain a regimen of healthy eating, regular physical activity, smoking cessation, and moderate alcohol consumption, as well as to maintain a consistent sleep pattern [62,63,64]. These lifestyle modifications are crucial in the management of T2DM, yet adherence to such interventions often falls short. Despite the potential for adverse effects and the development of resistance, oral hypoglycemic agents remain the most potent weapons in the fight against T2DM. The pharmacological armamentarium for the treatment and intervention of T2DM includes a range of oral hypoglycemic agents such as sodium-glucose transport protein 2 (SGLT2) inhibitors, biguanides, thiazolidinediones, and GLP-1 receptor agonists, as well as insulin [65,66,67]. Additionally, certain traditional Chinese medicines, such as Kudzu root, ginseng, Huanglian, and Huangqin, have been utilized [68,69,70]; however, the precise mechanisms of action for these traditional remedies in T2DM are not fully understood. Consequently, their application is generally discouraged in clinical settings.

In recent scholarly pursuits, the pharmacological properties of BAs have been a subject of rigorous investigation, yielding significant insights into their therapeutic potential, particularly in the context of T2DM. BAs, naturally occurring compounds of animal origin with historical usage in traditional Chinese medicine, have demonstrated efficacy in the management of this metabolic disorder, a phenomenon attributed to their interaction with the FXR receptor [59]. Despite sharing a common steroid nucleus, BAs exhibit variances in their physical attributes and biological activities. Contemporary research suggests that their role is not predominantly in the facilitation of lipid transport and absorption, but rather as sophisticated signaling entities. These compounds are capable of activating distinct signaling cascades via the FXR receptor, thereby enhancing glucose tolerance, augmenting insulin sensitivity, and improving energy metabolism [71]. The potency of BAs in engaging the FXR receptor is hierarchically ranked as follows: CDCA exhibits the highest efficacy, followed by LCA and DCA, which are comparable in their activity, and finally CA, with the least activation potential [72].

**Figure 5:**
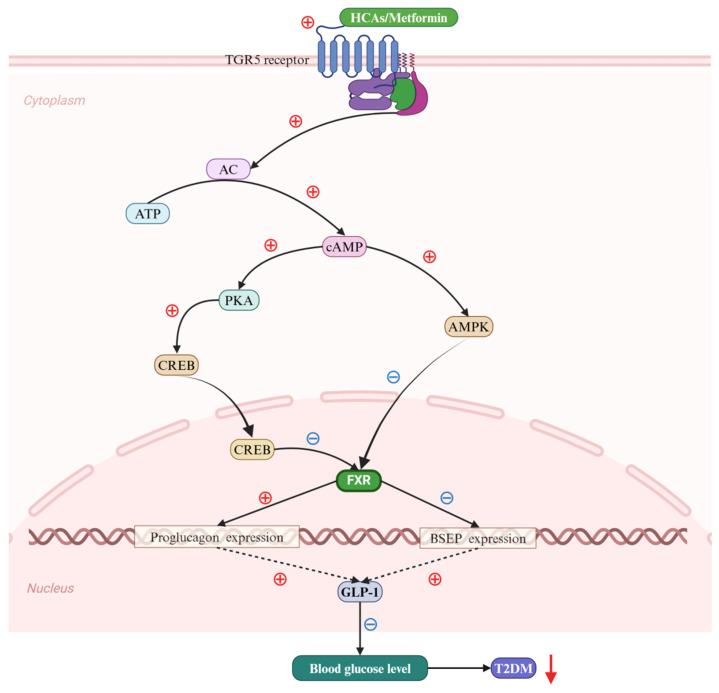
Bile acids (BAs) regulate Type 2 diabetes mellitus (T2DM) via the farnesoid X receptor (FXR). Hence, hyocholic acids (HCAs) and metformin potently activate the takeda G protein-coupled receptor 5 (TGR5), facilitating the production of cyclic adenosine monophosphate (cAMP) from adenosine triphosphate (ATP) under the catalytic action of adenylate cyclase. The elevated cAMP levels subsequently lead to the phosphorylation of both protein kinase A (PKA) and the cAMP response element-binding protein (CREB) (solid black arrows). This results in the phosphorylated CREB translocating into the nucleus, thereby initiating the nuclear import of CREB. Additionally, cAMP serves to activate the expression of MAP kinase (MAPK) (solid black arrow). The conjunction of phosphorylated CREB and MAPK targets the activation of FXR within the cellular nucleus (solid black arrow and black ray arrow). The FXR, in turn, enhances the expression of proglucogen and suppresses that of bile salt export pump (BSEP) genes (solid black arrows), which collectively contribute to a significant up-regulation of glucagon-like peptide 1 (GLP-1) expression (black dotted arrows). Ultimately, this cascade effectively reduces the progression of T2DM by decreasing blood glucose levels (down-solid red arrow). Abbreviations: ATP, adenosine triphosphate; BAs, bile acids; BSEP, bile salt export pump; cAMP, cyclic adenosine monophosphate; CREB, cAMP response element-binding protein; FXR, farnesoid X receptor; HCAs; hyocholic acids; GLP-1, glucagon-like peptide 1; MAPK, MAP kinase; PKA, protein kinase A; T2DM, type 2 diabetes mellitus; TGR5; takeda G protein-coupled receptor 5. Note: the symbol ⊕ indicates an increase, whereas ㊀ signifies a decrease.

The composition of BAs varies significantly across different animal species, each exhibiting unique physiological functions. For instance, the BA profile in humans is distinct from that in mice and pigs. In humans, the BA pool is predominantly hydrophobic, consisting of approximately 40% CA, CDCA, and 20% DCA [73]. In mice, the BA pool is highly hydrophilic, comprising about 50% CA, 50% T-αMCA, and T-βMCA [61]. In contrast, the BA pool in pigs is highly hydrophobic, consisting of approximately 75% HCAs [74]. Pigs are unique mammals with remarkable resistance to certain metabolic diseases, such as T2DM, NAFLD, and CVD [75,76,77]. Recent studies have revealed a correlation between obesity and T2DM and lower serum concentrations of HCAs, based on an analysis of 1107 samples (including 610 males and 497 females) from the Shanghai Obesity Research Group [78]. Hepatic cholesterol acids (HCAs) are potent regulators of blood glucose homeostasis, and have therapeutic potential for T2DM. These compounds act on the endocrine L cells of the colon and rat alveolar macrophages, activating the TGR5-inducing cyclic adenosine monophosphate (cAMP)–protein kinase A (PKA)–cAMP response element-binding protein (CREB) signaling pathway, and inhibiting the FXR receptor [79]. This action upregulates the expression of the proglucagon gene, promoting the generation and secretion of GLP-1 and effectively regulating blood glucose levels. Additionally, metformin, an effective medication for T2DM, can inhibit the expression of the BA transporter bile salt export pump (BSEP/ABCB11) by activating cAMP-PKA and cAMP-AMPK pathways mediating via TGR5 [80,81,82,83]. This method of BA participation is achieved by decreasing the expression of the FXR receptor, which in turn reduces blood glucose levels by increasing the secretion of GLP-1, thereby effectively controlling the progression of T2DM (Figure 5).

## 5. BAs Regulate Cancer via the FXR

Cancer stands as a leading global cause of mortality. Data from the American Cancer Society (ACS) and the National Cancer Institute (NCI) highlight that the predominant risk factors for cancer incidence encompass tobacco and smoking, alcohol intake, sedentary lifestyles, obesity, unhealthy dietary practices, excessive sun exposure, radiation exposure, viral infections, and various chemical contaminants [84]. In the year 2020, cancer accounted for nearly one million fatalities, representing approximately two-thirds of all deaths [85,86,87,88]. Notably, one-third of cancer-related deaths can be attributed to tobacco use, a high body mass index, alcohol consumption, an insufficient intake of fruits and vegetables, and physical inactivity [89]. The complexities associated with cancer treatment have persistently been a focal point of concern for the scientific community.

**Figure 6:**
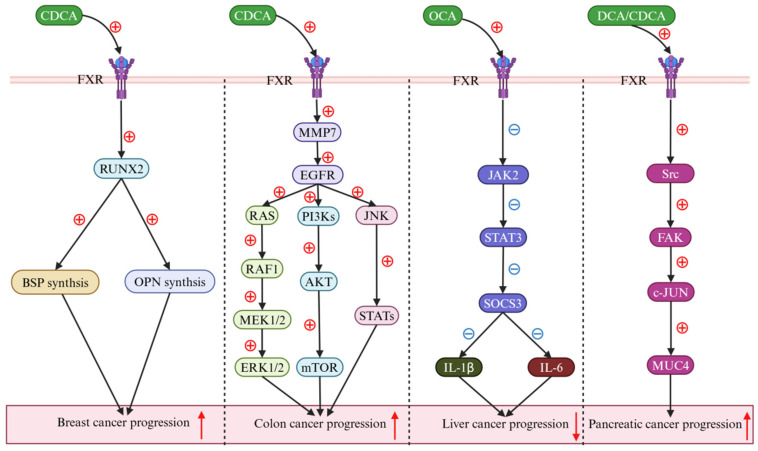
Bile acids (BAs) regulate cancers via the farnesoid X receptor (FXR). In breast cancer research, chenodeoxycholic acid (CDCA) has potently augmented runt-related transcription factor 2 (RUNX2) levels by triggering farnesoid X receptor (FXR) activation (down-solid black arrow), subsequently enhancing the synthesis of both bone sialoprotein (BSP) and osteopontin (OPN) (down-solid black arrow), thereby promoting the progression of the disease (up-solid red arrow). In colon cancer, CDCA markedly enhanced the expression levels of both matrix metalloproteinase 7 (MMP7) and the epidermal growth factor receptor (EGFR) (down-solid black arrow). Initially, the activated EGFR was found to promote the progression of colon cancer by engaging the Ras-Raf1-MAPK kinase 1/2 (MEK1/2)–extracellular signal-regulated kinase 1/2 (ERK1/2) signaling cascade (down-solid black arrow). Thereafter, it was revealed to contribute to the disease’s advancement through the phosphatidylinositol 3-kinase (PI3K)–protein kinase B (AKT)–mammalian target of rapamycin (mTOR) pathway (down-solid black arrow). Additionally, the activation of the EGFR was observed to facilitate the progression of colon cancer by modulating the c-Jun N-terminal kinase (JNK)–signal transducer and activator of the transcription (STATs) signaling pathway (down-solid black arrow). In liver cancer research, obeticholic acid (OCA) has been found to reduce interleukin-1β (IL-1β) and interleukin-6 (IL-6) secretion, thereby decelerating cancer progression (down-solid black arrow), which is attributed to the inhibition of the Janus kinase 2 (JAK2)–signal transducer and activator of transcription 3 (STAT3)–suppressor of cytokine signaling 3 (SOCS3) signaling pathway induced by FXR activation (down-solid black arrow). In pancreatic cancer, both deoxycholic acid (DCA) and CDCA markedly activated the FXR, orchestrating the Src-focal adhesion kinase (FAK)–c-JUN-mucin 4 (MUC4) signaling pathway (down-solid black arrow), and thereby contributing to the progression of pancreatic cancer (up-solid red arrow). Abbreviations: AKT, protein kinase B; BAs, bile acids; BSP, bone sialoprotein; CDCA, chenodeoxycholic acid; DCA, deoxycholic acid; ERK1/2, extracellular signal-regulated kinase 1/2; EGFR, epidermal growth factor receptor; FXR, farnesoid X receptor; FAK, focal adhesion kinase; IL-1β, interleukin-1β; IL-6, interleukin-6; JAK2, janus kinase 2; JNK, c-Jun N-terminal kinase; MMP7, matrix metallo proteinase-7; MEK1/2, MAPK kinase 1/2; MUC4, membrane mucin-4; mTOR, mammalian target of rapamycin; OCA, obeticholic acid; PI3K, phosphatidylinositol 3-kinase; RUNX2, transcription factor runt-related protein 2; SOCS3, suppressor of cytokine signaling-3; STATs, signal transducer and activator of transcriptions; STAT3, signal transducer and activator of transcription 3. Note: the symbol ⊕ denotes an upregulation, while the symbol ㊀ indicates a downregulation.

BAs, a category of metabolites synthesized within liver cells and subsequently released into the gastrointestinal tract, serve a pivotal role in the emulsification of dietary fats. Beyond this emulsifying capacity, BAs also function as crucial bioactive compounds within the body. Studies have illuminated that the FXR receptor, a receptor for BAs, exhibits differential expression across various cancer types [90]. Notably, it is overexpressed in kidney, thyroid, interstitial, pancreatic, esophageal, gastric, thymic, and lung cancers, yet is underexpressed in hepatocellular, colorectal, and prostate cancers [91]. The FXR’s influence on cancer cell invasion, metastasis, and angiogenesis confers diverse pathogenic roles. Absil et al. demonstrated that in the human breast cancer cell line MDA-MB-231, CDCA complexes with the FXR receptor and activates it, thereby initiating the translation of the transcription factor runt-related protein 2 (RUNX2) and the synthesis of bone sialoprotein (BSP) and osteopontin (OPN), which facilitate the progression and development of chest wall tumors [92]. Peng et al. revealed that, in the human colon cancer cell line HT-29, CDCA engages and activates the FXR receptor, leading to the upregulation of matrix metalloproteinase-7 (MMP-7), which in turn releases the epidermal growth factor receptor (EGFR) and triggers the activation of RAS/RAF/MAP kinase–ERK kinase (MEK)/extracellular signal-regulated kinase (ERK), phosphatidylinositol 3-kinase (PI3K)/protein kinase B (AKT)/mammalian target of rapamycin (mTOR), and Janus kinase (JAK)/signal transducer and activator of transcriptions (STATs) signaling pathways, thereby promoting the proliferation of HT-29 cells and accelerating colon cancer progression [93]. Attia et al. observed that, in HepG2, Huh7, and SNU-449 liver cancer cell lines, by targeting and activating FXR receptor, OCA inhibits the JAK2-STAT3 signaling pathway, downregulates the expression of the suppressor of cytokine signaling-3 (SOCS3), and consequently reduces the production and expression of cytokine interleukin-1β (IL-1β) and cytokine interleukin-6 (IL-6), effectively blocking the dissemination and progression of liver cancer [94]. Joshi et al. have uncovered a significant finding in the field of pancreatic cancer research. Their study indicates that DCA and CDCA, when bound to and activated by the FXR receptor, trigger the translation of the Src family of protein tyrosine kinases (SFKs), focal adhesion kinase (FAK), and c-Jun. This activation results in the upregulation of membrane mucin-4 (MUC4) expression, which plays an important role in the proliferation, survival, metastasis, and chemoresistance of pancreatic tumors [95]. These findings suggest that BAs, through their interaction with the FXR receptor, can modulate various signaling pathways and exert distinct pharmacological effects [96,97,98,99]. This discovery not only deepens our understanding of pancreatic cancer pathogenesis, but also paves the way for the development of novel BA-based therapeutic strategies and further exploration into the intricate relationship between BAs and cancer (Figure 6).

## 6. Conclusions and Outlook

The FXR receptor, a BA-activated nuclear receptor, plays a pivotal regulatory role, primarily in the liver and intestine. Activation of the FXR receptor initiates a diverse array of signaling cascades that profoundly influence the initiation and progression of various diseases [37]. These pathways include the nuclear factor erythroid 2-related factor 2 (NRF2), the fibroblast growth factor 15/19 (FGF-15/19)–fibroblast growth factor receptor 4 (FGFR4), the PKA-CREB, the Wnt/β-catenin, and the endothelin 1 (ET-1) signaling systems. When BAs and their derivatives bind to FXR, they activate it, thereby exerting significant pharmacological effects [69,100]. These effects are particularly notable in the context of metabolic disorders, such as NASH, NAFLD, and T2DM, where they help to rectify metabolic imbalances and mitigate disruptions in liver and intestinal function [66,70,101].

Through conducting in-depth explorations of the interplay and underlying mechanisms between the FXR receptor and BAs in metabolic regulation, recent scholarly investigations have revealed that the activation of the FXR receptor by BAs notably mitigates lipid peroxidation. This occurs through the upregulation of pivotal ferroptosis gatekeepers, including glutathione peroxidase 4 (GPX4), ferroptosis suppressor protein 1 (FSP1), the peroxisome proliferator-activated receptor α (PPARα), stearoyl-CoA desaturase 1 (SCD1), and the long-chain fatty acyl CoA synthetase 3 (ACSL3) [102]. As a specialized metabolic byproduct of gut microbiota, BAs have been identified to exert significant pharmacological effects, particularly in relation to the gut–liver axis, which is mediated by the FXR receptor. Furthermore, it has been observed that individual BAs exhibit varying degrees of binding affinity and efficacy in activating the FXR receptor, with the order of potency being CDCA > DCA > LCA > CA > other BAs [103]. In the current study, it has been discovered that certain BAs can facilitate the metastasis of cancers. For instance, BAs activate the FXR receptor, thereby promoting the metastasis of non-small cell lung cancer (NSCLC) by mediating the JAK2/STAT3 signaling pathway through the transactivation of IL-6ST and IL-6 genes [104]. In colon cancer cells, excessively high levels of BAs induce FXR expression, which promotes the progression of colorectal cancer in the post-initiation phase via the colonic mucosa, leading to a multitude of harmful effects, such as DNA oxidative damage, inflammation, and hyperproliferation [105]. Liu has identified that CA, DCA, and CDCA significantly activate the FXR receptor, resulting in the increased expression of BTB and CNC homology 1 (BACH1), glutathione (GSH) synthetase, and GPX4 in gastric cancer cells of HGC-27 and MKN-45 in a ferroptosis-dependent manner [106].

Chinese medicines have garnered increased global attention in the wake of the COVID-19 pandemic, largely due to their extensive clinical application, spanning thousands of years. These traditional remedies have demonstrated a range of therapeutic benefits, which are thought to be mediated through various signaling pathways such as NF-κB, PI3K-Akt-mTOR, p38-MAPK, Wnt-β-catenin, PI3K-Akt-GSK-3β, and MyD88 [107,108,109,110,111]. These effects encompass antioxidation, anti-aging, hypolipidemic and hypoglycemic properties, as well as their roles in radioprotection, anti-tumor activity, diabetes management, Alzheimer disease treatment, neuroprotection, and immune system enhancement [112,113]. A substantial body of research has demonstrated a correlation between the pharmacological properties of Chinese medicinal compounds and the FXR-BAs metabolic pathway. Tang’s study revealed that Lusisnthriding, derived from dendrobium, can stimulate the activation of FXR, thereby mitigating hepatic steatosis in mice on a high-fat diet by suppressing the expression of fatty acid synthesis genes, such as Srebp1c, Fas, Scd-1, Dgat1, and Lpin1. Baitouwengtang (BTWT) has been shown to effectively alleviate DSS-induced ulcerative colitis (UC) by modulating the gut microbiota and BAs through the FXR receptor and TGR5-signaling pathways [114]. Additionally, fucogalactan sulfate (FS), isolated from Laminaria, has been observed to inhibit the expression of the FXR receptor in the liver and reduce the expression of cholesterol 7α-hydroxylase (CYP7A1) and human sterol 12a-hydroxylase (CYP8B1), potentially decreasing BA synthesis and lipid absorption in mice with humanized dyslipidemia via the FXR-FGF19 signaling pathway [115]. This research facilitates the discovery and utilization of the role of the FXR receptor in the advancement of traditional Chinese medicine.

BAs-FXR receptor research has witnessed significant advancements in recent years, elucidating the pivotal role of BAs in governing physiological processes, such as metabolism, inflammation, and cell proliferation [116]. Investigations have discerned that the activation of FXR by BAs orchestrates metabolic regulation in the liver, intestines, and adipose tissue, mitigates inflammatory responses, and curbs the growth of tumor cells [117]. Nonetheless, the field of BAs-FXR receptor research involves several challenges. Firstly, the intricate biological conversion and metabolic pathways of BAs in vivo necessitate more comprehensive exploration of their nuanced metabolic mechanisms [118]. Secondly, the interplay between the BAs-FXR signaling pathway and other pathways remains elusive, demanding further investigation into its regulatory dynamics [119]. Furthermore, the development and screening of both BAs-FXR agonists and antagonists must surmount challenges related to bioavailability, toxicity, and adverse effects [120].

Future research on BAs-FXR should focus on several key aspects. Firstly, a thorough investigation into the mechanisms by which BAs function in physiological and pathological processes, with a particular emphasis on their roles in metabolism, inflammation, and the onset and progression of tumors [121]. Secondly, the development of novel BAs-FXR agonists and antagonists, enhancing their bioavailability and selectivity, while reducing toxicity and side effects [122]. Thirdly, exploring the interplay between the BAs-FXR signaling pathway and other pathways, thereby offering novel therapeutic strategies for diseases [123]. Lastly, studying the potential of the BAs-FXR signaling pathway as a therapeutic target for specific conditions, such as non-alcoholic fatty liver disease, type 2 diabetes, and cancer [124]. In summary, the research on BAs-FXR holds vast potential for application, promising to deliver innovative strategies and medications for the treatment of a multitude of diseases.

## Data Availability

No datasets were generated or analyzed during the current study.
